# An estimate of carbon emissions from 2004 wildfires across Alaskan Yukon River Basin

**DOI:** 10.1186/1750-0680-2-12

**Published:** 2007-12-19

**Authors:** Zhengxi Tan, Larry L Tieszen, Zhiliang Zhu, Shuguang Liu, Stephen M Howard

**Affiliations:** 1 SAIC, Contractor to USGS Center for EROS, Sioux Falls, SD 57198, USA; 2 USGS Center for EROS, Sioux Falls, SD 57198, USA

## Abstract

**Background:**

Wildfires are an increasingly important component of the forces that drive the global carbon (C) cycle and climate change as progressive warming is expected in boreal areas. This study estimated C emissions from the wildfires across the Alaskan Yukon River Basin in 2004. We spatially related the firescars to land cover types and defined the C fractions of aboveground biomass and the ground layer (referring to the top 15 cm organic soil layer only in this paper) consumed in association with land cover types, soil drainage classes, and the C stocks in the ground layer.

**Results:**

The fires led to a burned area of 26,500 km^2 ^and resulted in the total C emission of 81.1 ± 13.6 Tg (Tg, Teragram; 1 Tg = 10^12 ^g) or 3.1 ± 0.7 kg C m^-2 ^burned. Of the total C emission, about 73% and 27% could be attributed to the consumption of the ground layer and aboveground biomass, respectively.

**Conclusion:**

The predominant contribution of the ground layer to the total C emission implies the importance of ground fuel management to the control of wildfires and mitigation of C emissions. The magnitude of the total C emission depends on fire extent, while the C loss in kg C m^-2 ^burned is affected strongly by the ground layer and soil drainage condition. The significant reduction in the ground layer by large fires may result in profound impacts on boreal ecosystem services with an increase in feedbacks between wildfires and climate change.

## Background

Wildfires can result in losses of 15% – 35% of aboveground biomass and 37% – 70% of ground layers [1]. The pre-fire spatial variations in the C stocks in ground layers and the variations in the fraction of C consumed during burning both contribute to the uncertainty of C emission estimates [2]. *Neff et al. *[3] believe that the uncertainty is also attributed to the C density of deeper organic layers and the depth to which fires may reach.

It has been reported that soil drainage condition affects fire frequency and severity [4,5], vegetation recovery [6], and rates of organic matter decomposition [7] in the boreal region where thicker ground layer and higher soil C stocks are usually associated with poorer drained soils [4,5]. In other words, soil drainage conditions directly affect organic C accumulation as indicated by the thickness of the ground layer. On the other hand, soil drainage conditions determine the fractions of the ground layer consumed [8] and total fire emissions [4,9], even though fire weather conditions and seasonal thawing of soil or permafrost likely play an important role. For example, *Neff et al. *[3] estimated losses of up to 50% of ground layer C on poorly-drained sites, while *Stocks and Kauffman *[10] and *French et al. *[11] reported the losses of 60% to 90% on well-drained sites in Alaska. Because there are 40 – 60% of Alaska land consists of poorly drained soils [12], it is critical to understand how fire-induced C emissions are related to soil drainage classes.

There are a few studies to quantify ground fuels in Alaska [13,14], but there has been a lack of a spatially-explicit linkage of firescars to the information about ground layers and soil drainage conditions over the Alaskan boreal forests, while uncertainties associated with burning of ground layers which are commonly found in boreal forests and peatlands may be mainly responsible for the variation in fire-induced C emission estimate. According to *Kasischke et al*. [15], if only both ground fuel and burned area are considered to be responsible for the total variation in C emissions in the boreal forests, 63% are resulted from the uncertainty in ground fuels and 37% are attributed to the uncertainty in burned area, suggesting that the information about variations in ground fuels is essential for C emission estimation.

The year 2004 was the largest fire year in Alaska since 1950, and an estimate of C emissions for that year will help evaluate the contribution of wildfires in boreal forests to the global C budget. We took the Alaskan Yukon River Basin (YRB) as the study area because there was 83% of the total burned area across Alaska occurred within this area in 2004. The estimation of the C emissions from the wildfires in 2004 was based on the assumption that the level of fire-induced C release under a given fire weather condition depends on fire severity, land cover type, the thickness and C density of the ground layer, while these variables are supposedly associated with soil drainage conditions.

## Results

### Associations of firescars with soil drainage conditions

**Figure 1:**
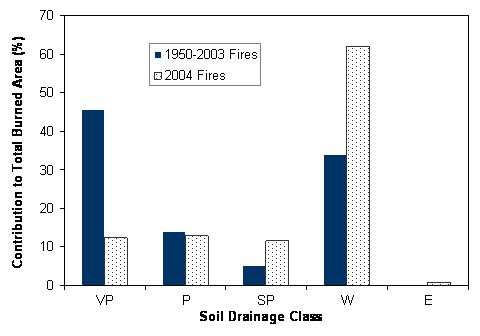
Associations of the firescars in 2004 with soil drainage conditions in comparison to previous firescars (VP, very poorly drained; P, poorly drained; SP, somewhat poorly drained; W, well drained; E: excessively drained. Both somewhat excessively and moderately well drained classes were not found in the firescars in 2004).

According to the Alaska STATSGO database [16], about 40% of all soils in the study area are classified as well drained class. However, 62% of the burned area in 2004 was associated with well drained class and only 25% with combined poorly and very poorly drained classes (Figure 1). In contrast, of the burned area between 1950 and 2003, 34% was associated with well drained class and 59% with the combined poorly and very poorly drained classes.

### Estimates of carbon emissions

The ground layer (referring to the top organic soil layer only in this study) within the burned area has a pre-fire average depth of 15 (± 4) cm. On average, the pre-fire C stock in the ground layer was 5.85 (± 1.02) kg C m^-2^, and the aboveground biomass (including the litter/lichen/moss layer) was about 2.23 kg C m^-2 ^(Table 1). Based on the scenarios of burn severity associated with a series of drainage classes, the total C loss from the fires in 2004 was estimated as 81.1 (± 1.36) Tg (Tg, Teragram; 1 Tg = 10^12 ^g), ranging from 68 to 96 Tg, 73% of which was attributed to the combustion of the ground layer. In other words, 37% of the aboveground biomass and 38% of the ground layer were lost from the fires. An average C release level of 3.06 kg C m^-2 ^burned was estimated for all burned area. The estimates for low and high burn severity scenarios would be 1.35 and 3.73 kg C m^-2 ^burned, respectively.

**Table 1:** Pre-fire carbon stock and carbon release from the fires in 2004 in the study area.

	Unit	Aboveground Biomass		Ground Layer (organic soil)	Total
				
		Tree/shrub/grass	Litter/ichen/moss		
Pre-fire	Kg C m^-2^	1.73 (0.53)^a^	0.50 (0.06)	5.85 (1.02)^b^	7.92 (1.42)
	T g C	45.8	13.3	155.0	214.1
Carbon Loss	Kg C m^-2^	0.52 (0.09)	0.31 (0.06)^a^	2.23 (0.69)	3.06 (0.70)
	TgC	13.8 (0.4)	8.2	59.1 (12.5)	81.1 (13.6)
	%	30	61	38	38
Contribution	%	17	10	73	100

^a ^The proportion (0.62) was assumed to be equivalent to areal portion of well-, and excessively well drained soil areas where whole litter/lichen/moss layer might be burned completely and no burning happened to poorly drained soils.

^b ^The average depth of organic (soil) is 15 cm. (The numbers in parentheses are the standard deviations of the means).

### Contributions of land cover types

**Figure 2:**
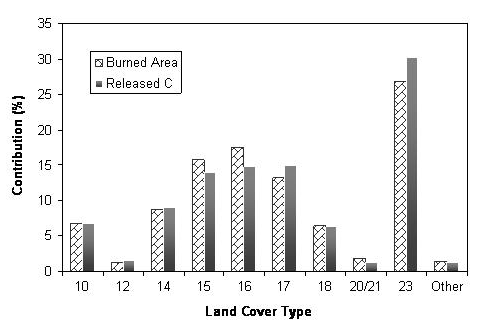
Contributions of each land cover type to total burned area and total C release (Note for land cover type: 10, tall shrub; 12, closed mixed forest; 14, spruce woodland/shrub; 15, open spruce/shrub/bog mosaic; 16, spruce broadleaf forest; 17, open & closed spruce forest; 18, mixed spruce forest mosaic; 19, closed spruce & hemlock forest; 20/21, 1990/1991 firescars; 23, tall & low shrub).

As illustrated in Figure 2, the land cover type *Tall & Low Shrub *(code 23) accounted for 27% of all burned area which was nearly three times its average distribution proportion across the study area, followed by the types *Spruce Broadleaf Forest *(code 16), *Open Spruce Forest/Shrub/Bog Mosaic *(code 15), *Open & Closed Spruce Forest *(code 17), etc. Correspondingly, the type *23 *made the greatest contribution to the total C emission (30%). The types *17, 16*, and *15 *contributed about 15% each.

## Discussion

The total C emission from the 2004 wildfires across the YRB is about 6.7 times the annual anthropogenic emissions (12.3 Tg C) from Alaska in 2003 [17], or equivalent to 61% of the annual biomass C photosynthesized over the study area if the average NPP was about 266 g Cm^-2 ^in 2004 [18].

Generally, our estimate of C emissions from the 2004 fire year is comparable with those reported for previous wildfire years in Alaska. For example, *Kasischke et al.*[19] reported an average C release of 2.88 kg C m^-2 ^from 1990/1991 fires. *Kasischke and Bruhwiler *[20] estimated a range of total C emissions from 1.77 to 3.72 kg C m^-2 ^burned for the 1998 boreal forest fires across the Western North America by setting three different burn severity class scenarios (low, medium and high) and assuming each class had a different set of fractions of C consumed. *Kasischke et al*. [21] also simulated a range of 1.17 to 4.22 kg C m^-2 ^burned from the 1990 boreal forest fires in the central YRB.

The consumed C fractions of both aboveground biomass and the ground layer are very close to the average values reported previously. The C density of pre-fire ground layer expressed as kg C m^-2 ^per cm depth of the ground layer, our estimate of 0.34 kg C m^-2 ^per cm depth is comparable to the 0.36 kg C m^-2 ^per cm depth derived from the data of *Kasischke et al. *[21], but higher than the 0.21 kg C m^-2 ^per cm depth reported by *Kane et al. *[22]. As presented in Table 1, 73% of all consumed C was contributed by the combustion of the ground layer. It has been realized that the consumed portion of the ground layer (or reduction in the depth by burning) depends on the burn severity and ground wetness at burning time [4,23,24]. Historical fire records indicate that about 20% more burned area was associated with well drained soils in 2004 than in 1990/1991. Again, the consumed fraction of aboveground biomass varies with vegetation type [21,25]. About 60% of all burned area in 2004 was associated with the shrub-related land cover types. However, the error associated with the Alaska vegetation class dataset could partially contribute to the uncertainty of the estimate due not only to the coarse spatial resolution but also to the quality of remote sensing data and interpretation. Note that the firescars data from the Alaska BLM Wildland Fire Dataset [26] were collected based on the reporting system. Such reporting systems may come up with uncertainties due to inaccuracy in determining perimeters and missing data, which consequently could contribute some to the uncertainty of our estimates.

Another important contributor to the uncertainty of the C release estimate is burn severity. Data are scarce for verifying burn severity of the Alaskan fires in 2004. As a case study, we examined the 1999's firescars in the Yukon-Charley National Preserve. We estimated the C release from the fires in 1999 based on the moderate burn severity scenario. We estimated C release from the 2004 fires again, using the burn severity map (with low, moderate and high classes) to define the consumed C fractions for aboveground biomass and ground organic layers. The burn severity map was generated using the differenced normalized burn ratio (dNBR) approach that is suggested by the U.S. Wildland Fire Leadship Council for the Monitoring Trends in Burn Severity project. No significant difference was observed, although the method using three burn severity classes resulted in a higher estimate than did the method with an assumption that all fires were moderate severity.

According to *French et al. *[11], large fire years usually lead to a high burn severity; and more burned area associated with well drained soils generally have a higher consumed C fraction of ground layers. Therefore, both together, as characterized by the 2004 wildfires across the YRB, certainly lead to a high C release. In this study we accounted for the contribution of the ground layer to the total C emission by spatially relating individual firescars to the pre-fire land cover types and also defining the consumed C fractions in association with both the soil drainage classes of the firescars and the C stocks in the ground layer. This can partially trade off some uncertainty associated with burn severity quantification. Note that the uncertainty of the estimate might be also attributed to the errors associated with the STATSGO database [16] from which the pre-fire C stocks of the ground layer were derived. Unfortunately, the soil database is the only data source available on the state scale even though its spatial resolution is not fine enough for the presented study. Meanwhile, the STATSGO approach ignored the fact that the upper part of the ground layer has a lighter C density than does the lower part (by about 20% to 30%), implying that the consumed C fraction and the total C emission based on the C density averaged for the ground layer were certainly overestimated. Assuming the C density of the upper part of the ground layer is 20% smaller than that of the lower part, the total C emission would be about 68.7 Tg and the contribution of the ground layer to the total C emission would be reduced to 70%. Such an estimate is close to that (70.5 Tg C) made by Ottmar et al. [27] in which they classified all fuels across the YRB into three FCCS fuelbed types (i.e. birch and aspen, black spruce, and willow/alder shrubland) to run their model CONSUME 3.0.

As indicated in Table 1, the ground layer along with the litter/lichen/moss layer was estimated to contribute as high as 83% to the total C emission (about 6 cm top organic soil layer was destroyed), implying the importance of ground fuel management to the control of wildfires and mitigation of C emissions. More importantly, the consequences and impacts of such predominant contribution can be profound on the boreal ecosystem services and future global change. First, about 77% of all C stock is stored in ground layers and soils in the whole boreal region, and the C emissions from historical wildfires in this region might have been largely underestimated by previous approaches. Second, large wildfires are associated with the boreal forests that have large areas of permafrost [28,29]. The fire-induced C emissions from the combustion of the ground layer could enhance degradation of permafrost. It has been documented that the fire- and warming-induced collapse of ecosystem structure and permafrost has damaged infrastructure, altered discharge pathways of surface water, disrupted native subsistence cultures, and aided the spread of invasive species [30,31]. An increase in the frequency and extent of boreal forest wildfires in response to the warming climate will lead to additional deterioration of boreal ecosystem functioning.

To more accurately predict potential C emissions from wildfires, additional pre- and post-fire sampling studies are needed to quantify ground layers over large areas for monitoring changes in both ground layers and permafrost with various wildfire regimes.

## Conclusion

The predominant contribution of ground organic layers to the total C emission implies the importance of ground fuel management to the control of wildfires and mitigation of C emissions. The magnitude of the total C emission depends on fire extent, while the C loss in kg C m-2 burned is affected strongly by ground organic layer and soil drainage condition. The significant reduction in the ground layer by large fires may result in profound impacts on boreal ecosystem services with an increase in feedbacks between wildfires and climate change.

## Methods

### Study area and firescars

**Figure 3:**
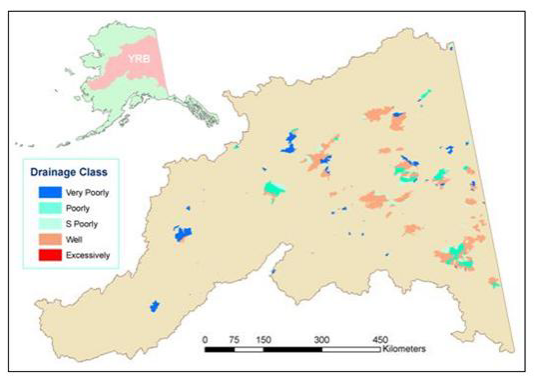
Yukon River Basin in Alaska and the distribution of firescars in 2004 in association with soil drainage classes.

The YRB in Alaska, or the interior Alaska (see Figure 3), covers an area of 502,451 km^2^. The forests in the study area have thick ground organic layers which usually consist of litter, lichen, mosses, and ground layers [32]. However, the Fuel Characteristic Classification System (FCCS) [27] considers the litter/lichen/moss layer on the ground as a part of the aboveground biomass (i.e., the aboveground biomass consists of trees (canopy, snags, and ladder), shrubs, grasses, dead wood, litter/lichen/moss layer on the ground); and the ground layer (or organic soil layer). The ground layer can be differentiated into the fibric (Oi), hemic (Oe), and/or sapric (Oa) horizons under the U.S. Soil Classification System. Both the litter/lichen/moss layer and the ground layer are particularly vulnerable to fire disturbance [11], which makes boreal forest burning differ from those in the temperate and tropical zones. Because the present study is closely related to wildfires and may be more interesting to fuel management, the C emissions from different fuel categories were estimated following the fuel classes of the FCCS in this study.

The historical wildfire records [26] show that about 35% of the study area experienced wildfires between 1950 and 2005. The wildfires in 2004 resulted in 102 firescars (larger than 40 ha each) as indicated by the polygons in Figure 3, and burned 26,500 km^2^. The ignorance of firescars smaller than 40 ha is because their proportion to the total burned area is insignificant [33].

### Algorithm for estimating C release from fires

Estimating C emissions from fires is a multi-step process. First, to determine the areas burned by relating firescars to land cover types; then determine C stocks associated with land cover types by spatially linking to the C stocks in ground layers from the Alaska STATSGO database [16]; finally, to estimate consumed and emitted C fractions for each land cover type and soil drainage class defined by *Rieger et al. *[34]. Consumed C fractions are defined for both aboveground biomass and ground layers which are associated with land cover type and soil drainage class, respectively.

The equation (1) modified by *French et al*. [35] from that of *Seiler and Crutzen *[36] was used to estimate the total C release (C_t_) from burning of both aboveground biomass and ground layers:

C_t _= A(C_a_β_a _+ C_g_β_g_)

where *A *is the total area burned (ha); *C*_*a *_is the average C density of aboveground biomass (kg C m^-2^), assuming the C fraction of the aboveground biomass is about 0.50 [11]; *β*_*a *_is the fraction of aboveground biomass consumed during a fire; *C*_*g *_is the C density (kg C m^-2^) of ground layers exposed to a fire, and *β*_*g *_is the fraction of the organic layers consumed by the fire.

### Land cover types in association with firescars

The 1990/1991 Alaska Vegetation/Land Cover map [37] used in this study was derived from the Advanced Very High Resolution Radiometer (AVHRR) data set, and the classification was developed using the phenology of a vegetation index (AVHRR/NDVI) collected during the 1991 growing season with 1 km spatial resolution. The land cover types associated with the firescars within the study area in 2004 are presented in Table 2.

**Table 2:** 1990–1991 land cover type inventory across the study area and areal percentage of each land cover type involved in the fires in 2004.

**Code**	**Land Cover type (total area is 502, 451 km^2^)**	**Area %**	**Area involved in the fires (%)**
1	Water	0.7	
2	Glacier & Snow	2.2	
3	Alpine Tundra	5.2	0.01
4	Dwarf Shrub Tundra	3.6	0.06
5	Tussock Sedge/Dwarf Shrub Tundra	1.1	0.81
6	Moist Herbaceous/Shrub Tundra	1.6	0.36
8	Low Shrub/Lichen Tundra	1.4	0.05
10	Tall Shrub	5.7	6.78
12	Closed Mixed Forest	0.1	1.26
14	Spruce Woodland/Shrub	3.9	8.80
15	Open Spruce/Shrub/Bog Mosaic	26.7	15.87
16	Spruce Broadleaf Forest	13.2	17.75
17	Open & Closed Spruce Forest	11.6	13.32
18	Mixed Spruce Forest Mosaic	10.2	6.56
19	Closed Spruce and Hemlock Forest	0.1	0.06
20/21	1990/1991 Firescars	3	1.28
23	Tall and Low Shrub	9.6	26.96

### Consumed C fractions of aboveground biomass and ground layers

The C magnitudes of aboveground biomass (*β*_*a*_) as defined by the FCCS for different land cover types in the study area were derived from the literature. The baseline C stocks (*C*_*g*_) of the ground layers involved in the fires consists only of the upper organic soil layers, and was derived from the Alaska STATSGO database [16] which has no information about the litter/moss layer. If there were no soil bulk density data available for calculating the C stock, then the bulk densities were estimated with the equation developed by *Adams *[38] as follows:

BD_ej _= 100/[(OM_mj_/0.22) + (100 - OM_mj_)/ρm]

where BD_ej _estimated bulk density for the j^th ^layer (g cm^-3^)

OM_mj _median value of OM concentration range (%)

ρm the bulk density with zero organic matter (usually using 1.60 (g cm^-3^) to replace ρm)

0.22 a conversion factor for OM to become a portion of total soil mass.

Meanwhile, the C pool of the litter/lichen/moss layer was estimated with assumptions as follows: (1) the average depth and C density of the litter/lichen/moss layer are supposed to be 4 cm and 138 g C m^-2 ^cm^-1^, respectively [21]; and (2) the depth of the litter/lichen/moss layer is assumed to vary with drainage classes and depths 2, 3, 5, 5, and 2 cm are assigned for very poorly drained, poorly drained, somewhat poorly drained, well drained, and excessively drained, respectively.

The fractions of C consumed during burning vary largely with individual fire events and vegetation types, ranging from 0.10 to 0.90 for a variety of different physiographic settings in Alaska [21,39]. A set of average fractions of C consumed which represent a range of burning conditions for the Alaskan boreal forests are summarized in Table 3.

**Table 3:** Carbon contents of aboveground biomass and ground organic layers and consumed C fractions related to burn severity levels in the Alaskan Yukon River Basin.

Category	Biomass Kg C m^-2^	Fraction consumed (β) with burn severity			Reference
			
		High	Moderate	Low	
Above ground Biomass	1.56				[40]
	2.30	0.33	0.23	0.12	[35]
	2.30		0.45		[19]
	2.09	0.50	0.34	0.14	[21]
Ground Layer (or Organic Soil)	9.00	0.56	0.36	0.18	[35]
	6.00		0.36		[19]
	6.89	0.62	0.45	0.27	[21]
Mean-above ground	2.06	0.42	0.34	0.13	
Mean-Organic soil	7.30	0.59	0.39	0.23	

In our study, we overlaid the GIS theme of soil drainage classes with the firescars theme and assigned the average fractions of C consumed (see Table 3) to individual firescars in reference to their land cover types and soil drainage classes as defined by the Alaska STATSGO. Soil drainage classes that were associated with the firescars included very poorly drained, poorly drained, somewhat poorly drained, well drained, and excessively drained. The C fractions of the ground layer consumed were assumed to be 0.25 for the very poorly drained, 0.30 for the poorly drained, 0.35 for the somewhat poorly drained, 0.45 for the well drained, and 0.60 for the excessively drained [3,4,8-11]. For the 1990/1991 land cover types named *1990/1991 firescars*, the land cover type of *shrub *was assumed to be the predominant vegetation succession [41] for the aboveground biomass estimation. For the firescars occurred between 1990 and 2003 which were reburned in 2004, we assumed that the baseline C stocks in the top 15 cm ground layer for estimating the 2004 burning emissions were reduced by 39% based on a moderate burn severity (see Table 3).

## Competing interests

The author(s) declare that they have no competing interests.

## Authors' contributions

ZT designed this study, prepared data, analyzed results, and drafted the manuscript. LLT and ZZ supervised this research and made comments on the manuscript formulation. SL gave technical support and critical thoughts on the manuscript finalization. SMH assisted in processing images for burning severity mapping. All authors read and approved the final manuscript.
